# *In situ* X-ray absorption and emission spectroscopy to understand the electron transfer–oxygen transfer reaction of vanadium polyoxomolybdate in a homogeneous medium

**DOI:** 10.1039/d6sc02170k

**Published:** 2026-05-14

**Authors:** Kamar Bendehiba, Santanu Sarmah, Estelle Pujol, Dominique Nkeuya, Dominik Neukum, Vera Truttmann, Dmitry E. Doronkin, Nuria Romero, Philippe Serp, Jan-Dierk Grunwaldt, Bidyut Bikash Sarma

**Affiliations:** a Université de Toulouse, Laboratoire de Chimie de Coordination (LCC), CNRS, INPT/UPS UPR 8241, 205 route de Narbonne 31077 Toulouse Cedex 4 France bidyut-bikash.sarma@lcc-toulouse.fr bidyutbikash.sarma@toulouse-inp.fr; b Institute of Catalysis Research and Technology, Karlsruhe Institute of Technology (KIT) Hermann-von Helmholtz Platz 1 76344 Eggenstein-Leopoldshafen Germany; c Institute for Chemical Technology and Polymer Chemistry, Karlsruhe Institute of Technology (KIT) Engesserstraße 20 76131 Karlsruhe Germany; d Institut Universitaire de France (IUF) 1 rue Descartes F-75231 Paris France

## Abstract

Electron transfer–oxygen transfer reactions of vanadium polyoxomolybdates have been investigated in the homogeneous catalytic oxidation of xanthene and biomass-derived compounds. At room temperature, we observed the formation of a stable adduct between the polyoxometalate and xanthene following the initial electron transfer in acetonitrile. The high energy resolved fluorescence detected X-ray absorption near edge structure spectroscopic investigations revealed that vanadium occupies a distorted octahedral position within a defect site in the polyoxometalate framework. *In situ* X-ray emission spectroscopy further revealed that, at first, vanadium is reduced from V^V^ to V^IV^ and there are changes in the coordination around the vanadium during the electron transfer with significant changes in the valence to core Kβ″ and Kβ_2,5_ lines. This fundamental investigation of molecular catalysis tackles some of the key questions regarding electron transfer–oxygen transfer reactions using state-of-the-art spectroscopic techniques.

## Abstract 2

The dynamic structural evolution of a polyoxometalate in different solvent media is demonstrated through the oxidation of xanthene in acetonitrile and polyols in a water + methanol mixture.

## Graphical abstract

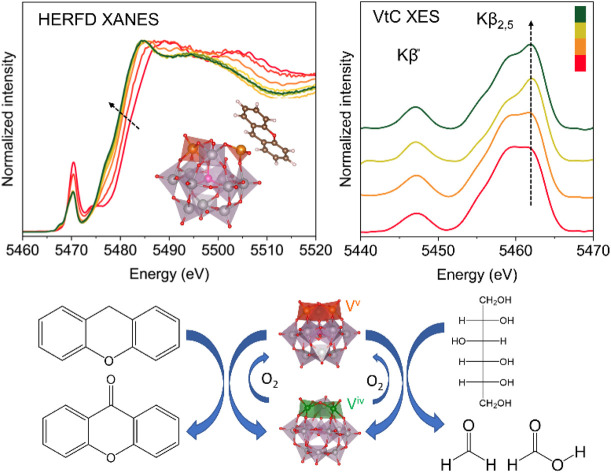

## Introduction

Polyoxometalates (POMs) are metal oxide clusters formed by a self-assembly process which usually takes place in highly acidic media.^1–3^ The metals that constitute POMs are typically in their highest oxidation state (Mo^VI^, V^V^, W^VI^ and others) and display octahedral coordination geometry.^2,4^ POMs have been shown to be active catalysts for acid^5^ and supported^6^ catalysis, electrochemical reactions,^7^ biomass conversion^8^ and many other catalytic reactions.^9–13^ Despite the full coordination of the metal centers, POMs are excellent candidates for electron transfer (ET), proton transfer (PT) and oxygen transfer (OT) reactions.^14–16^ Some of the best known examples of POMs are the vanadium and molybdenum containing POMs known as phosphovanadomolybdates, H_3+*x*_PV_*x*_Mo_12−*x*_O_40_ (*x* = 1–6), which are Keggin type polyoxometalates. These POMs have demonstrated high catalytic activity for oxidative transformations, though it is notable that this activity is highly solvent dependent. For example, in acetonitrile, H_5_PV_2_Mo_10_O_40_ (PV2) was found to be active for the electron and oxygen transfer processes, which constitute the oxidation of electron rich aromatic compounds such as anthracene or xanthene.^17^ During the OT between PV2 and the aromatic compounds, a Mars–van Krevelen (MvK) mechanism prevails, which is widely known for oxidation reactions over metal-oxide based solid catalysts.^18^ The mechanism was proved using ^18^O-labeled molecular oxygen, which was incorporated into the PV2 structure, and finally transferred to the aromatic compounds during oxidation.

A major focus of POM research lies in their ability to catalyze C–C cleavage and C–H activation reactions, with the influence of the solvent emerging as a central topic in the field. Khenkin *et al.* showed that PV2 is active for C–C bond cleavage of primary alcohols and vicinal diols with sulfolane ((CH_2_)_4_SO_2_) as the solvent.^19^ This reaction is very slow or does not proceed at all in other polar aprotic solvents such as acetonitrile or nitromethane. ^18^O-labeled experiments show that there is a direct OT between PV2 and the alcohol. The C–C bond cleavage is reported not to be rate-determining; it was found that protonation of the alcohol determined the rate of the reaction. Sarma *et al.* further showed that C–C bond cleavage of polyols, such as glucose and fructose, can occur in a water/methanol mixture as the solvent to produce the desired formaldehyde and formic acid, which subsequently form methyl formate, dimethoxymethane, methoxymethanol and methylene glycol.^20^ Using water as a solvent resulted in 50% selectivity toward CO_2_ formation, whereas a mixture of water and methanol led to more than 90% selectivity towards desired products and less than 10% CO_2_ formation, which was confirmed by others.^21–23^ Moreover, the re-oxidation of PV2 is faster in a water/methanol mixture compared to pure water and the reaction is very slow in pure methanol. Additionally, when 80% sulfuric acid was used as the solvent, the polyols reacted at 60 °C compared to water where the reaction takes place above 100 °C.^20^ However the re-oxidation of PV2 is challenging and does not occur at all in highly acidic media. To re-oxidize PV2, the authors used bulk electrolysis, which led to the protons and electrons stored in PV2 being released as hydrogen. The authors also showed that the use of sulfuric acid as a solvent facilitates POM-catalyzed C–H bond activation for molecules such as toluene or benzene. Toluene and substituted toluene were able to form benzaldehydes^24^ whereas phenol was formed from benzene.^25^ The authors identified a stable benzene radical with the help of electron paramagnetic resonance (EPR) spectroscopy and all these reactions were shown to proceed *via* ET–OT steps.

**Fig. 1:**
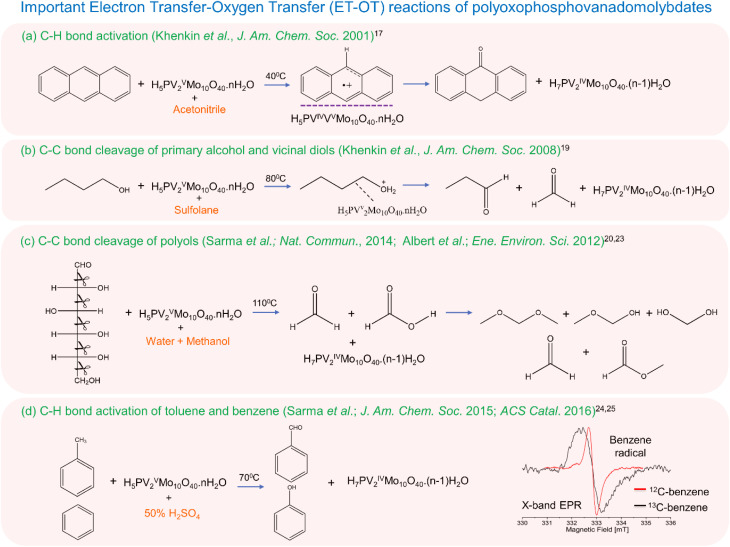
Some important PV2-catalyzed ET–OT reactions in different solvents. (a) Anthracene in acetonitrile,^17^ (b) alcohol and diols in sulfolane,^19^ (c) polyols in a water/methanol mixture,^20^ (d) methyl arenes and benzene in aqueous sulfuric acid.^24,25^

To understand the fundamental steps behind such ET–OT reactions, Kaminker *et al.* used W-band EPR spectroscopy to uncover the speciation of reduced PV2 during ET. Here they identified two species.^26^ One is a vanadyl (VO^2+^)-like species, whose fraction is around 30–35%, and the rest is [PV^V^Mo_10_O_39_]^6−^. The authors found that the VO^2+^ species is not situated within the POM cluster whereas the other vanadium remains intact within the POM. According to their findings, PV2 exists as [PV^V^Mo_10_O_39_]^6−^[V^IV^O^2+^], which is claimed to be the reactive form. Another study by Tiwari *et al.* showed that dissolution of PV2 in 50% aqueous H_2_SO_4_ generates two reactive pervanadyl VO_2_^+^ ions, which are highly active for the oxidation of p-xylene to *p*-methylbenzaldehyde.^27^ A summary of some important PV2-catalyzed ET–OT reactions as discussed above is presented in Fig. 1, alongside the relevant reaction conditions.

From this analysis of the literature, it is clear that ET–OT processes involving PV2 are highly dependent on the reaction medium, which could be linked to the re-oxidation of PV2 by molecular oxygen. The observation that the reactive VO_2_^+^ species can be formed from PV2 during the reaction raises questions such as: (a) what is the original state of PV_*x*_ in the solution? (b) Does the substrate bind to vanadium *via* a V–C bond during the reaction? (c) How do different reaction media influence the reactivity of the PV_*x*_?

In order to tackle these questions, we have synthesized a series of phosphovanadomolybdates H_3+*x*_PV_*x*_Mo_12−*x*_O_40_ (*x* = 1–3) and compared their catalytic activity for two different ET–OT reactions: (a) C–H activation for xanthene in acetonitrile and (b) C–C bond cleavage of vicinal diols in a 1 : 1 mixture of water and methanol. The progress of the reaction between xanthene and PV_*x*_ in acetonitrile has been monitored by *in situ* X-ray absorption spectroscopy (XAS) and emission spectroscopy (XES) (so-called photon-in/photon-out) at the vanadium K-edge to identify the evolution of vanadium species during the ET–OT reaction. Special attention has been paid to solvent POM interactions throughout this analysis.

## Results and discussion

### Synthesis and characterization

**Fig. 2:**
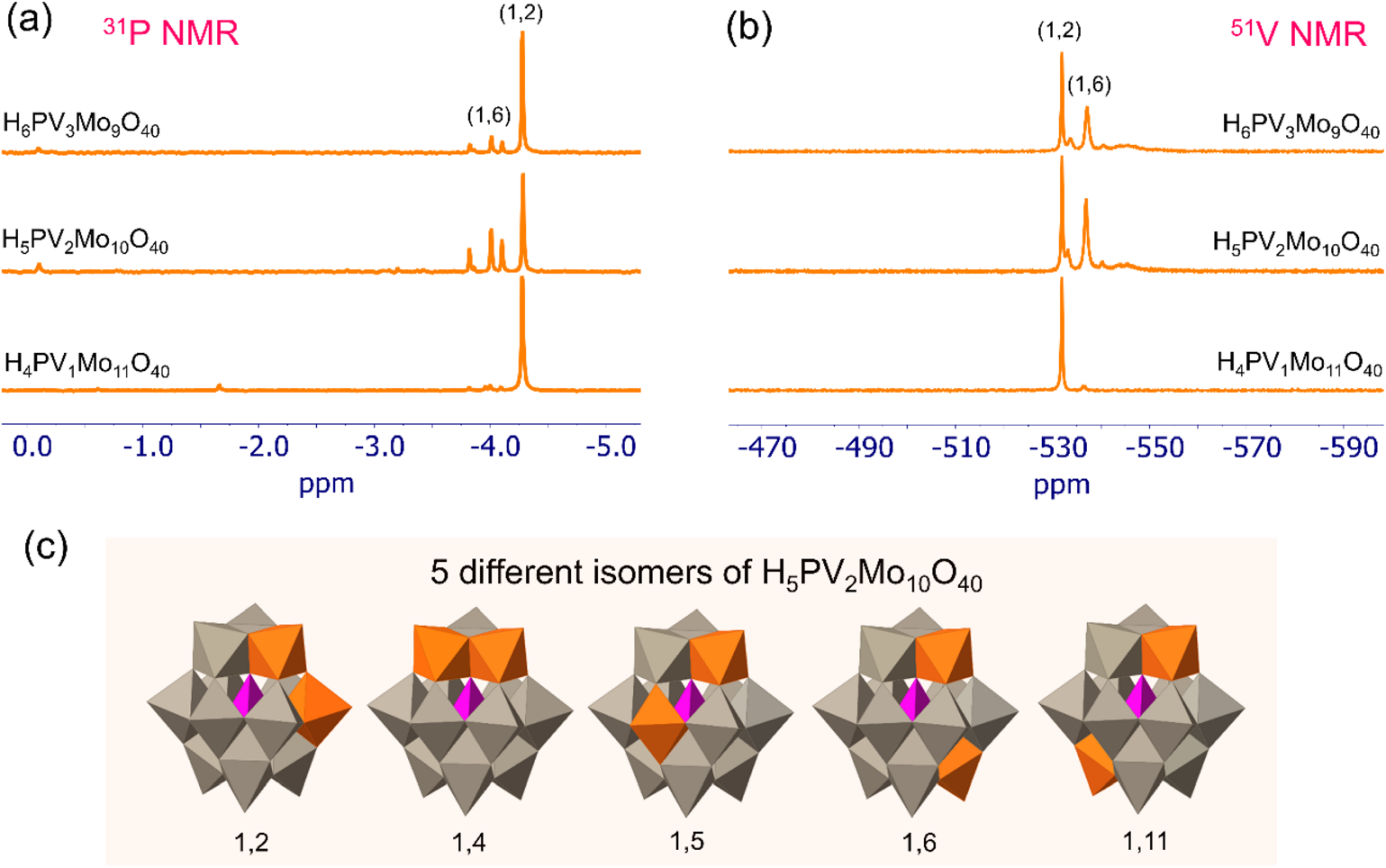
Characterization of the synthesized polyoxophosphovanadomolybdates PV1, PV2 and PV3: (a) ^31^P NMR, (b) ^51^V NMR, (c) polyhedral model structures of the five isomers of PV2 where the two vanadium atoms occupy five different places (1,2), (1,4), (1,5), (1,6) and (1,11). Color codes are orange: vanadium; gray: molybdenum; purple: phosphorous. The abundance of these isomers depends on the solvent used and pH of the medium as reported in the literature.^29,30^

The phosphovanadomolybdates H_3+*x*_PV_*x*_Mo_12−*x*_O_40_ (*x* = 1–3) were synthesized by an earlier reported procedure.^28^ Details are given in the experimental part of the SI (page S3). H_4_PV_1_Mo_11_O_40_, H_5_PV_2_Mo_10_O_40_ and H_6_PV_3_Mo_9_O_40_ are denoted as PV1, PV2 and PV3, respectively. These catalysts were characterized by ^31^P and ^51^V NMR. The ^31^P NMR spectra in Fig. 2(a) reveal that there is a predominant peak (−4.3 ppm) for PV1 whereas there are 5 peaks in the case of PV2, which is due to the presence of five different isomers as shown in Fig. 2(c). For PV3, there is also the possibility of formation of isomers. As during the synthesis of PV1 small amounts of PV2 or PV3 may also form, traces of these compounds were also detected by NMR. The ^51^V NMR spectra showed a similar trend as shown in Fig. 2(b).

**Fig. 3:**
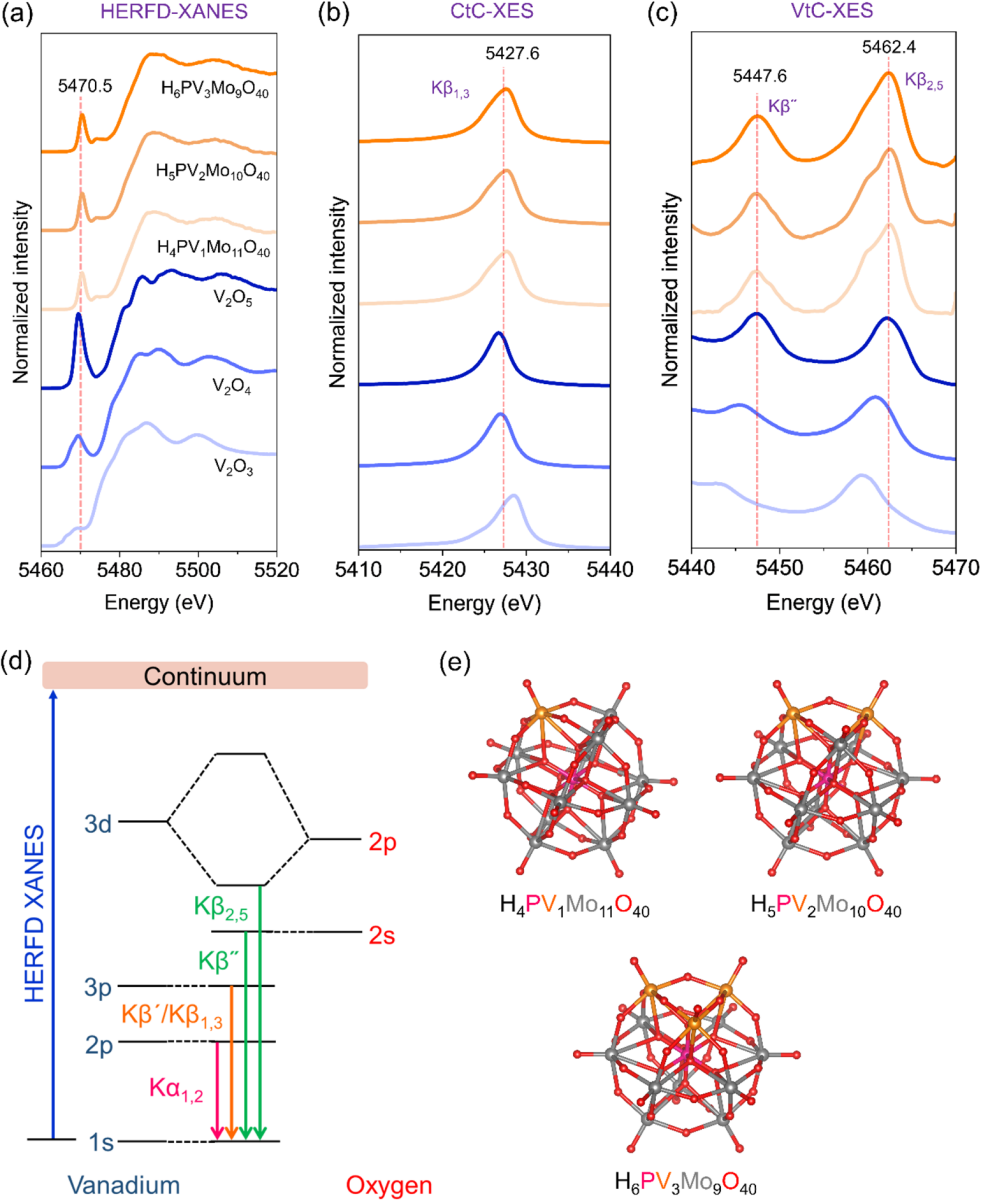
Characterization of the polyoxovanadates PV1, PV2 and PV3 with reference oxides V_2_O_5_, V_2_O_4_ and V_2_O_3_ at V K-edge. (a) HERFD-XANES, (b) CtC-XES (Kβ_1,3_), and (c) VtC-XES (Kβ″ and Kβ_2,5_), (d) schematic representation of the origin of different XES spectral features, and (e) ball and stick model structure of PV1, PV2 and PV3. The dotted lines are a guide to the eye. CtC and VtC stand for valence to core and core to core. The PV1, PV2 and PV3 were measured by dissolving in acetonitrile and continuous flow.

In order to reveal the oxidation state and ligand environment around vanadium, high energy-resolution fluorescence detected X-ray absorption near edge structure (HERFD-XANES) spectra^31,32^ were collected at the vanadium K-edge together with the reference vanadium oxides as shown in Fig. 3(a). The details of the experiment are given in the SI (page S3). The HERFD-XANES spectra correspond to the excitation of an electron from the core as depicted in Fig. 3(d). From the position of the rising edge in Fig. 3(a), it is clear that the vanadium is in the oxidation state of +5 (*cf.* V_2_O_5_ reference). However, there are two pre-edge peaks at 5470.5 and 5474 eV due to weak symmetry forbidden transitions, which indicate that the vanadium is not in an octahedral coordination but rather in a distorted symmetry. The peak position and the pre-edge features are comparable to the literature reports on vanadium containing catalysts.^33–35^

To investigate the coordination between vanadium and oxygen atoms, we further collected XES spectra at the vanadium K-edge as shown in Fig. 3(b and c). The Kβ XES region shows features related to the ligand environment.^36–40^ During XES, ionization of a 1s core electron of vanadium takes place by high energy incident X-rays, followed by monitoring the emission of photons during electron decay to fill the 1s hole as shown in Fig. 3(d). Kβ XES is usually sensitive to the oxidation and spin states, surrounding ligand identity, hybridization and protonation as was shown both experimentally and by theoretical calculations for Fe and other metals.^39,41–45^

The Kβ_1,3_ emission line is a result of electric dipole allowed core-to-core 3p–1s transition and it is weakly sensitive to the ligand environment. However, it can be used to derive the oxidation and spin states.^34,46^ In Fig. 3(b), there is a clear shift observed towards lower energy among the metal oxides (V_2_O_3_, V_2_O_4_ and V_2_O_5_). However, in all the POMs (PV1, PV2 and PV3), there are two features observed: one sharp feature at 5427.6 eV and a shoulder in the left, which may be an indication of two types of vanadium-oxygen species.

The valence to core (VtC) XES spectra (Fig. 3(c)) showed two prominent features, Kβ″ and Kβ_2,5_, that are related to the ligands surrounding the metal as schematically shown in Fig. 3(d). The Kβ″ feature presents the cross-over transition from a molecular orbital of ligand character and, in our case, it is oxygen as vanadium is surrounded by O atoms. The intensity of the Kβ″ feature depends on the V–O bond, and especially the coordination number and interatomic distance. In the present study, all the POMs show this feature to be similar to the one of V_2_O_5_, meaning that the V–O bond is comparable to the one of V_2_O_5_. On the other hand, the position of Kβ_2,5_ depends on the oxidation state of vanadium. When vanadium is more oxidized, the emission line is shifted to higher energy. This is illustrated by the position of the Kβ_2,5_ feature following the order V_2_O_3_ < V_2_O_4_ < V_2_O_5_.

**Fig. 4:**
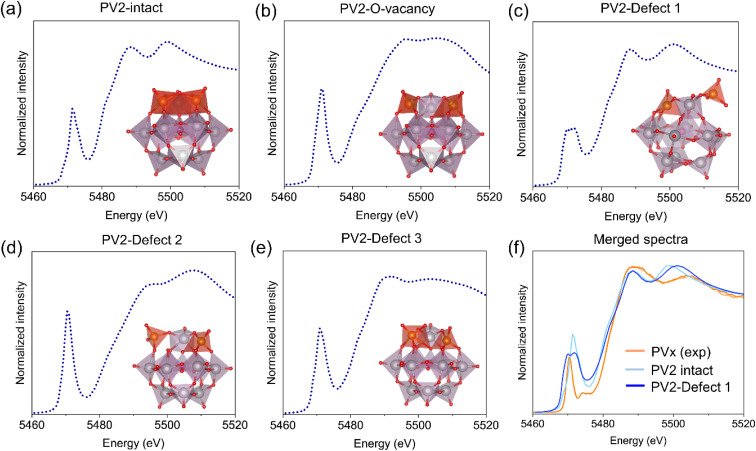
Theoretical XANES spectra of PV2 at V K-edge: (a) intact PV2, (b) PV2 with O-vacancy and (c–e) PV2 with different defect sites with the corresponding polyhedral model representation, (f) comparison of the experimental (PV_*x*_) and theoretical (PV2 intact and PV2-defect 1) XANES spectra. The DFT optimized model structures were taken from ref. 45.

Based on these results, we took the model of PV2 and hypothesized possibilities of three defective structures: defect 1, where vanadium is bonded to the original structure by one metal–oxygen bond; defect 2, where vanadium is bonded to the original structure by two metal–oxygen bonds and defect 3, where vanadium is bonded to the original structure by three metal–oxygen bonds as reported in the literature.^47^ Additionally, we considered two structural models of PV2: one containing an oxygen vacancy as a defect site, and one intact structure in which all vanadium–oxygen bonds remain fully coordinated within the POM framework. We then carried out theoretical calculation of the XANES spectra by using FEFF10 code^48,49^ as shown in Fig. 4(a)–(e). Out of all these structures, PV2 with defect 1 showed two pre-edge peaks and most of the matching features as observed in the experimental HERFD-XANES spectra shown in Fig. 4(f). The PV2 intact does not show the double pre-edge feature and the PV2 with oxygen vacancy also show a very strong pre-edge feature. This indicates that the vanadium occupies a defect site within the POM framework. The relative intensities of the two-pre-edge feature is still quite unbalanced as there is still possibility of having mixed species. It is also important to mention here that the lattice water present in the crystal structure of PV*x* is highly important and without it the PV*x* structure is prone to disintegrate.^50^

### Catalytic tests of electron transfer–oxygen transfer (ET–OT)

**Fig. 5:**
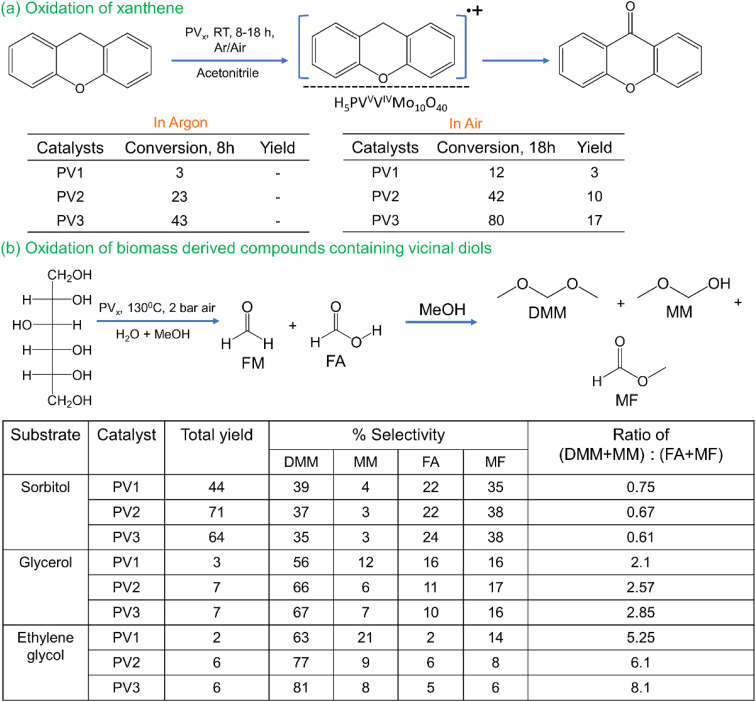
Catalytic tests of (a) oxidation of xanthene and (b) oxidation of biomass derived molecules with polyphosphovanadates (PV_*x*_). For product quantification, a gas chromatograph coupled with a flame ionization detector and ^1^H NMR were used for reactions (a) and (b), respectively.

The details of the catalytic tests are provided in the experimental part of the SI (page S4). For the catalytic tests, we selected two sets of reactions under different conditions: (a) oxidation of xanthene in acetonitrile and (b) oxidation of biomass derived molecules in a water/methanol mixture. The reason for choosing these two reactions is because they proceed under different conditions. The ET–OT reaction between xanthene and PV_*x*_ can occur at room temperature, whereas for diols/polyols, it takes place at elevated temperature (>100 °C). Biomass is one of the abundant sources for synthetic fuel and other valuable platform molecules,^51^ and the use of heteropolyacids for the conversion of cellulosic biomass to formic acid has been already reported in the literature.^52^ However, a detailed mechanistic investigation is needed in order to understand the ET and OT reactions. In our investigation, we have used PV1, PV2 and PV3 as catalysts and compared their reactivity for both reactions. The catalytic results are summarized in Fig. 5.

#### ET–OT reaction of xanthene with POMs

We carried out the reaction of xanthene with PV_*x*_ in acetonitrile under various conditions in a Fisher Porter tube. Under argon, we observed maximum conversion of 43% with PV3 after 8 hours with a corresponding color change from orange to green; however, no product was detected in all cases (Fig. 5(a)). By changing the atmosphere to air, we started observing xanthone as a product as shown in Fig. 5(a). This indicates that there is an initial PV_*x*_–xanthene adduct formed after the electron transfer from the substrate to vanadium (color change from orange to green). Then, in the presence of oxygen, the polyoxometalate is re-oxidized, leading to the formation of xanthone. Upon increasing the temperature to 80 °C, 98% conversion and 72% xanthone yield were obtained as shown in Table S1 of the SI (page S6). This indicates that there is a resting state of the PV_*x*_–xanthene radical and it is a stable adduct at room temperature for a prolonged period of time. The reaction of the PV*x* with xanthene at 80 °C in the presence of air led to nearly full conversion and >95% selectivity after 24 hours as shown in Table S1 of the SI. The conversion follows the trend of PV3 > PV2 > PV1 according to the increase in vanadium content in the POM.

**Fig. 6:**
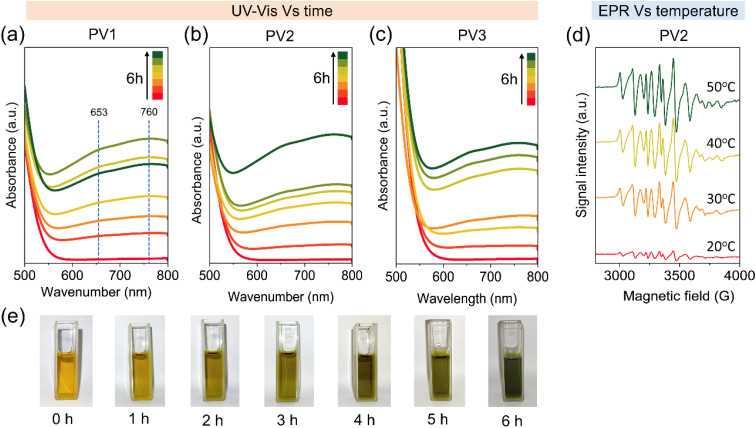
(a)–(c) Time-dependent UV-vis spectra of an equimolar mixture of PV1, PV2, and PV3 with xanthene in acetonitrile, (d) temperature-dependent EPR spectra of an equimolar mixture of PV2 and xanthene in acetonitrile, and (e) pictures of the cuvette with reaction mixtures at different times. The dotted line is a guide to the eye.

UV-vis spectroscopy was carried out with PV*x* (0.2 mmol) and xanthene (0.2 mmol) at room temperature and the spectra were collected every 1 hour over the period of 6 hours as shown in Fig. 6(a)–(c). The spectra showed the emergence of two peaks at wavelengths of 630 and 750 nm, which increased in intensity over time. The peak at 653 nm is due to the ion pair complex between the xanthene cation radical and reduced PV*x* as xanthone in acetonitrile shows an absorption maximum at 631 nm as reported in the literature,^53^ and the peak at 750 nm is due to the reduced PV^V^V^IV^Mo_10_O_40_^6−^.^54^ Khenkin *et al.* also observed a similar absorption peak at 650 nm which they assigned to the ion pair complex between PhSMe^•+^ and the reduced H_5_PV^V^V^IV^Mo_10_O_40_.^55^ Strong absorption below 550 nm comes from the ligand to metal charge transfer (LMCT). The corresponding color change of the solution is shown in Fig. 6(e). Additionally, we carried out EPR spectroscopy at different temperatures (20, 30, 40 and 50 °C) by taking the reaction mixture of PV*x* (0.2 mmol) and xanthene (0.2 mmol) in acetonitrile after reacting for 3 hours. We observed a strong signal of vanadium at 20 °C which gradually became more intense upon increasing the temperature to 50 °C as shown in Fig. 6(d). As ^51^V has a nuclear spin of 7/2, an eight-component hyperfine structure is expected from the dipole–dipole interaction between the magnetic moment of the ^51^V nucleus and the unpaired electron (*s* = 1/2) of V^4+^ ions. However, we did not observe any xanthene radical as it may be highly unstable in acetonitrile as explained in the literature.^17^

**Scheme 1:**
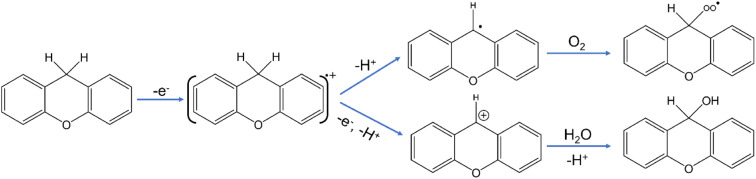
Proposed mechanism of subsequent electron and proton transfer between PV*x* and xanthene.

Based on these results, we propose that the reaction between xanthene and PV*x* leads to the formation of a cationic radical intermediate which occurs due to direct electron transfer between the xanthene and PV*x* as shown in Scheme 1. The xanthene cation radical then forms either a neutral xanthene radical (*via* deprotonation) or a xanthene cation (*via* subsequent electron and proton transfer). The presence of oxygen further leads to formation of peroxo intermediates, which ultimately decompose to form xanthen-9-ol and finally xanthone. Alternatively, the presence of water can lead to nucleophilic reaction between the xanthene cation and water resulting in xanthen-9-ol. The oxygen or the water required at the last step can come from the participation of lattice oxygen (*via* the Mars–van Krevelen type mechanism) or water that is always present in the crystal lattice of PV*x*.”

#### ET–OT reaction of diols/polyols with POMs

In the second set of reactions, we carried out the C–C bond cleavage of diols and polyols such as sorbitol, glycerol and ethylene glycol with POMs in a water/methanol mixture as a solvent at 130 °C (Fig. 5(b)). This reaction does not proceed in acetonitrile. The three substrates were chosen based on the fact that they contain different number of primary and secondary alcohols. Sorbitol can be obtained from the hydrogenation of cellulose,^56^ glycerol is a valuable byproduct in biodiesel production,^57^ and ethylene glycol can be produce from biomass components^58,59^ and, most importantly, from depolymerization of polyethylene terephthalate (PET).^60^ Upon oxidation of these molecules, the primary alcohols are supposed to form an aldehyde (formaldehyde, FM) and the secondary alcohols will form an acid (formic acid, FA). As FM tends to oxidize to FA in the presence of oxygen, we have used a mixture of water and methanol (50 : 50) to trap the FM intermediate as dimethoxymethane (DMM), which is considered to be a cleaner synthetic fuel.^61^ However, due to the presence of water, we also observed the formation of methoxymethanol (MM) and traces of methanediol (MD), as also reported in the literature.^20^ The ^1^H and ^13^C NMR spectra of the reaction mixture are shown in Fig. S1 and S2 of the SI (pages S7 and S8). Additionally, FA can be esterified to yield methyl formate (MF) under these conditions, which is beneficial as FA tends to decompose to CO_2_ in water.

As among the substrates, the ratio of primary to secondary alcohol is the highest in the case of ethylene glycol; the ratio of yield of DMM + MM + MD to (FA + MF) will follow the order of ethylene glycol > glycerol > sorbitol. From these results it can also be inferred that the cleavage of the C–C bond occurs at the secondary alcohol, as the cleavage of the primary alcohol is very slow (we achieved a maximum of 6% yield for ethylene glycol). Even though the maximum total yield of the products was found to be 71% for sorbitol, this can be further improved by increasing the reaction temperature and pressure of air. For example, by increasing the temperature to 150 °C and the air pressure to 5 bar, up to 85% of the total yield of the products could be obtained.

The next question is whether it is a homogeneously catalyzed reaction or if there is involvement of solid vanadium species. We therefore tested different vanadium oxides (V_2_O_5_, V_2_O_4_, V_2_O_3_) and molybdenum oxides (MoO_2_) under the same reaction conditions. The results are shown in the SI (Table S2, page S9). Since at the end of the reaction all the reaction mixtures were colored (except MoO_2_), we filtered out the solid catalysts and ran another test with the filtrate by adding additional amounts of sorbitol. We observed an enhanced yield of all products, suggesting that the reaction is catalyzed by homogeneous vanadium species that leach from the solid vanadium oxides. The formation of the molecular species of vanadium from the vanadium oxides was confirmed by ^51^V NMR spectroscopy as shown in Fig. S3 (page S10) of the SI.

We then carried out stability and recyclability tests of the PV_*x*_. The ^31^P NMR spectra before and after the reaction did not show significant differences as shown in Fig. S4 of the SI (page S11). The major peak at −4.28 ppm is consistent with the preservation of the intact structures of PV1, PV2, and PV3. The recyclability tests, run for 5 consecutive cycles, also did not show any significant decline in catalytic activity and any degradation of the PV2 catalyst as shown in the ^31^P NMR spectrum in Fig. S5 (page S12) of the SI.

Another vital question is why the reactivity of the POM changes based on the solvent used. One answer is that it forms different reactive vanadium species in different solvents. For example, Tiwari *et al.*^27^ showed that in a 50% sulfuric acid–water mixture, PV2 forms two reactive pervanadyl VO^2+^ species which are capable of oxidizing the C–H bond of methyl arenes. Therefore, we carried out a series of ^31^P and ^51^V NMR experiments in water, methanol and acetonitrile with freshly prepared PV1, PV2 and PV3. For these measurements, we took single crystals of these catalysts obtained after crystallization in water. The ^31^P and ^51^V NMR spectra are shown in Fig. S6 and S7 (page S13 and S14) of the SI. It is evident that various species are formed in different solvents due to interaction of the solvent with the POMs. In stronger coordinating solvents such as methanol and acetonitrile more peaks were observed in comparison to water. This indicates that such species could play a major role during reaction and explains why the reaction of PV_*x*_ with different substrates relies on the reaction medium. Krueger *et al.* showed that the presence of methanol poisons the active site of PV2 and significantly slows down the reaction between PV2 and glycolaldehyde.^30^ When we carried out the reaction between PV2 and methanol at 150 °C under 2 bar of air, we observed the formation of DMM and traces of FA and the color of the reaction mixture was green at the end of the reaction. This suggests that methanol can bind to the vanadium center and stabilize the V^IV^ oxidation state. The exact role of the solvent in each of the reactions discussed in this manuscript is currently under investigation and will be reported in a follow-up work.

### *In situ* XES investigation

**Fig. 7:**
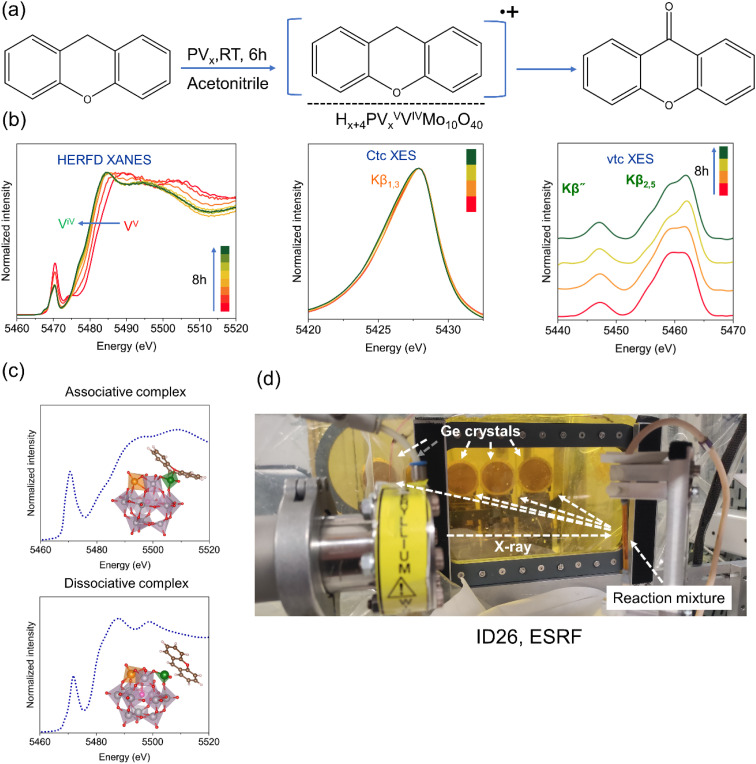
*In situ* XES studies during reaction of xanthene with PV3. (a) Scheme of the oxidation reaction of xanthene to xanthone, (b) time-resolved *in situ* HERFD-XANES, XES (Kβ_1,3_) and background subtracted VtC-XES (Kβ˝, Kβ_2,5_) spectra, (c) theoretical XANES spectra of the PV2 with associative and dissociative complexes with xanthene and (d) experimental setup at ID26, ESRF, Grenoble, France.

*In situ*/*operando* spectroscopy is vital to understand the dynamic evolution of active sites during the reaction.^62,63^ We carried out *in situ* HERFD-XANES and XES experiments during the reaction between xanthene and PV3 at room temperature for 10 hours in the presence of air as shown in Fig. 7. The experimental setup at the ID26 beamline of the European Synchrotron Research Facility (ESRF) is shown in Fig. 7(d). As discussed earlier, during the reaction, there is a gradual change in color from orange to green, which indicates that there is an electron transfer between PV3 and xanthene. When we followed the V K-edge HERFD-XANES spectra (Fig. 7(b)) several changes were noticed. First, the rising edge shifted towards lower energy (indicated by an arrow), which means that vanadium is reduced. Secondly, the pre-edge feature at 5474 eV disappeared over time, while the feature at 5470 eV decreased in intensity. This may be attributed to the formation of a more symmetric coordination environment around V atoms (*e.g.*, octahedral symmetry). To confirm this, we took two DFT optimized model structures from the literature^47^ representing potential interactions. After the ET, vanadium: (1) is strongly bonded to the xanthene *via* a vanadium-carbon/oxygen bond or (2) weakly interacts with xanthene as a radical pair. Both are possible and during the reaction, the vanadium-xanthene radical pair adduct slowly dissociates as also observed experimentally. The simulation of the XANES spectra by taking these two models fits well with the experimental spectra as shown in Fig. 7(c) as well as Fig. S8 (page S15).

There is also a shift of the Kβ_1,3_ emission line towards higher energy (Fig. 7(b)), confirming that vanadium is changing from a higher to lower oxidation state. We also observed changes in the valence to core (VtC) Kβ″ and Kβ_2,5_ lines, which is an indication of the change in the oxygen ligand orbital. For example, Safonova *et al.* showed that when a V–O bond is replaced by a V–N bond, there is a clear change in the VtC-XES spectra indicated by a several eV shift of the Kβ″ line to higher energy; the less electronegative carbon would cause an even stronger shift.^35^ However, in our case, if a V–C bond forms, the process is very fast leading to a long living species. Therefore, we could not observe a clear change to indicate the formation of a V–C bond.

**Fig. 8:**
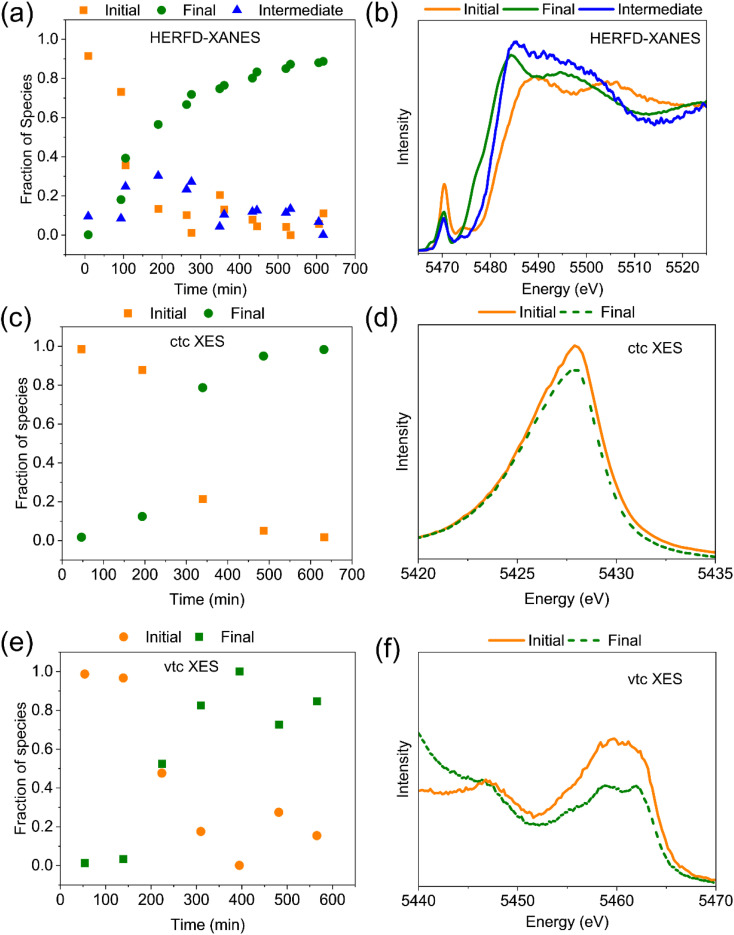
(a), (c) and (e) Fraction of components derived from MCR ALS of the HERFD-XANES, CtC-XES, and VtC-XES (not background subtracted) spectra of PV3 with xanthene. (b), (d) and (f) show the respective spectral components extracted by the MCR-ALS refinement procedure. Note that the absence of background subtraction and different normalization procedures required for the robust MCR-ALS results in different appearance of the extracted spectral components as opposed to the data treated by standard XAS/XES procedures shown in Fig. 7.

To uncover the kinetics of the transformation of V species during the reaction between xanthene and PV3 we applied Multivariate Curve Resolution-Alternating Least Squares (MCR-ALS) to the measured HERFD-XANES dataset recorded during the reaction of xanthene with H_6_PV_3_Mo_10_O_40_ as shown in Fig. 8. Three spectral components could be identified as constituents of the dataset, one similar to the starting and end state of V species, and one for the intermediate species having a maximum with approx. 30 mol% concentration at approx. 3 hours on stream (Fig. 8(a)). The initial spectral component, representing the starting PV3 state, has a similar shape to the others with two maxima in the XANES region (Fig. 8(b)) but with a higher pre-edge. As noted above, this suggests a higher coordination number, possibly corresponding to a change from five-coordinate to octahedral symmetry. The decreased pre-edge intensity is also consistent with a lower oxidation state (reduction of V^5+^ to V^4+^).^64^ As seen from the concentration profiles, the initial PV3 state is transformed into two states at the same time, both with a similar low pre-edge, possibly signifying the same octahedral coordination of V species. While the molar fraction of the final state spectrum keeps constantly increasing, the fraction of the intermediate state spectral component slowly decreases in favor of the final component whose molar fraction reaches 90% after 10 hours on stream. The main difference between the end state and the intermediate spectral components is the rising edge position. For the intermediate it is closer to the rising edge of the starting state (close to V^5+^), whereas the final state spectrum corresponds to V^4+^. Hence, after the reaction of PV3 with xanthene is initiated, at first the coordination number of V is increased (pre-edge intensity decreased) without actual reduction of V^5+^, which occurs in the second step with a much slower rate. The MCR-ALS performed at the VtC and CtC Kβ_1,3_ spectra could only resolve two components as show in Fig. 8(c) and (d). We believe that the two species corresponding to starting and intermediate HERFD-XANES components have similar oxidation and spin states and are therefore indistinguishable during evaluation of the XES datasets. However, we observed similar temporal trends in both the VtC and CtC Kβ_1,3_ spectra qualitatively fitting also to the trend in conversion of the sum of the initial and intermediate HERFD-XANES components to the final spectral state (*i.e.* V^5+^ to V^4+^), even considering the large scatter in the VtC XES derived trend due to the low sensitivity (error on the order of 30 rel.% due to low photon counts originating from the low probability of the corresponding electronic transitions).

## Conclusion

**Fig. 9:**
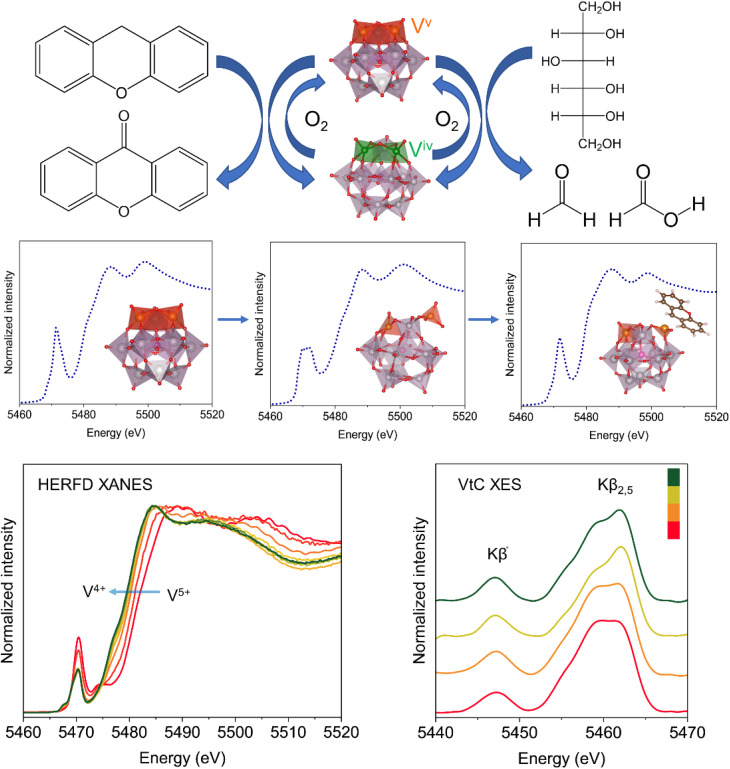
Summary of the ET–OT reaction between xanthene, polyols and polyoxophosphovanadomolybdates. The theoretical XANES spectra with the model structure of PV3 and the *in situ* HERFD-XANES and VtC-XES spectra.

In this work, we have synthesized and characterized vanadium containing polyoxometalates and investigated the ET–OT reaction with the help of *in situ* X-ray absorption and emission spectroscopy complemented by theoretical prediction of the model structures. The HERFD-XANES spectroscopic investigation showed that the vanadium is in a distorted octahedral coordination and present in a defect site, which allows it to participate in the reaction. The ET–OT reactions of the polyoxometalate have been investigated under two reaction conditions as summarized in Fig. 9. Among the polyoxophosphovanadomolybdates, H_5_PV_2_Mo_10_O_40_·nH_2_O (PV2) showed excellent catalytic activity for the oxidation of xanthene to xanthone and for the conversion of vicinal diols to formaldehyde and formic acid. The ET between polyoxometalate and xanthene takes place at room temperature in acetonitrile to form a stable adduct as evident from the UV-vis spectroscopy and EPR results, which dissociates only at elevated temperature. The *in situ* investigation complemented by theoretical simulation of the XANES spectra suggests that: (a) the vanadium center is already in a distorted state in acetonitrile and as the reactions proceeds, vanadium is reduced from the V^V^ to V^IV^ state; and (b) after the ET, vanadium is less distorted than in its original state due to stable adduct formation between POM and xanthene. Photon-in/photon-out spectroscopy in terms of HERFD-XANES and Kβ XES showed that the vanadium is reduced during the reaction as evident from the Kβ_1,3_ line and there are changes in the coordination around the vanadium during the ET as shown by the changes in the Kβ″ and Kβ_2,5_ features, which originate from the electronic transition from the ligand (O) to the core-hole on the excited V ion. The MCR-ALS analysis performed on the *in situ* HERFD-XANES spectra during the reaction of PV3 with acetonitrile suggests the presence of three different components during the reaction. On the other hand, the ET between polyoxometalates and vicinal diols occurs at a high temperature in a mixture of water and methanol as solvent which shows the role of solvent during such processes. The NMR investigation showed that solvents such as methanol coordinate to the vanadium center already at room temperature which requires further in-depth investigation. These results open up a new avenue in molecular catalysis to investigate such ET–OT reactions with techniques that go beyond the state of the art and pave the way for understanding such fundamental processes. Therefore, it will serve as a benchmark and inspire many others to use such techniques for answering challenging questions in homogeneous catalysis.

## Author contributions

The manuscript was written through contributions of all authors. BBS conceptually designed all the experiments, carried out the synthesis of catalysts and experimental work at the synchrotron, data analysis and theoretical calculation of the spectra. KB, EP, DN, and SS carried out the catalytic tests, optimization and qualitative and quantitative analysis under the supervision of BBS and NR. DN, VT and DD assisted in the experimental work at the synchrotron and data analysis. PS and JDG provided all the infrastructure required for the studies and contributed to the manuscript writing. All the authors participated in the discussion and reviewed the final version of the manuscript.

## Conflicts of interest

The authors declare no conflict of interest.

## Supplementary Material

SC-017-D6SC02170K-s001

## Data Availability

All the raw data related to synthesis, catalysis and *in situ* investigation will be made available to anyone upon request to the corresponding author. Supplementary information (SI): experimental protocols of catalyst synthesis, characterization (UV-vis, EPR spectroscopy) catalytic tests results, and NMR spectra of crude product mixtures. See DOI: https://doi.org/10.1039/d6sc02170k.
