# Supplementary material for: In situ X-ray absorption and emission spectroscopy to understand the electron transfer–oxygen transfer reaction of vanadium polyoxomolybdate in a homogeneous medium
Source: Chem Sci. 2026 May 14;17(26):13042–54. doi: 10.1039/d6sc02170k (PMC13202792; doi:10.1039/d6sc02170k)
Supplement: SC-017-D6SC02170K-s001 [file SC-017-D6SC02170K-s001.pdf]

This journal is © The Royal Society of Chemistry 2026

Electronic Supplementary Information

***In situ* X-ray absorption and emission spectroscopy to understand the electron transfer-oxygen transfer reaction of polyoxometalate in homogeneous medium**

Kamar Bendehiba[a], Santanu Sarmah,[a] Estelle Pujol[a], Dominique Nkeuya[a], Dominik Neukum[b], Vera Truttmann[b,c], Dmitry E. Doronkin[b,c], Nuria Romero[a], Philippe Serp[a,d], Jan-Dierk Grunwaldt[b,c], Bidyut Bikash Sarma*[a]

[a]Université de Toulouse, Laboratoire de Chimie de Coordination (LCC), CNRS, INPT/UPS, UPR 8241, 205 route de Narbonne, 31077 Toulouse Cedex 4, France

[b]Institute of Catalysis Research and Technology, Karlsruhe Institute of Technology (KIT), Hermann-von Helmholtz Platz 1, 76344 Eggenstein-Leopoldshafen, Germany

[c]Institute for Chemical Technology and Polymer Chemistry, Karlsruhe Institute of Technology (KIT), Engesserstraße 20, 76131 Karlsruhe, Germany

[d] Institut Universitaire de France (IUF), 1 rue Descartes, F-75231 Paris, France

Email: bidyut-bikash.sarma@lcc-toulouse.fr; bidyutbikash.sarma@toulouse-inp.fr

Table of contents

Contents	Pages
**Experimental**	**S3-5**
Table S1: ET-OT reaction between PV_x and xanthene	**S6**
^{1}H NMR of product mixture after reaction of PV2 with sorbitol	**S7**
^{13}C NMR of product mixture after reaction of PV2 with sorbitol	**S8**
Table S2: Catalytic tests for oxidation of sorbitol	**S9**
^{51}V NMR spectra of filtered product mixture after reaction of V_2O_5 and V_2O_4 with sorbitol	**S10**
^{31}P NMR spectra before and after reaction between PV_x and sorbitol	**S11**
^{31}P NMR spectra after first and five cycles of reaction between PV_x and sorbitol	**S12**
^{31}P NMR spectra of PV1, PV2 and PV3 in water, methanol and acetonitrile	**S13**
^{51}V NMR spectra of PV1, PV2 and PV3 in water, methanol and acetonitrile	**S14**
Theoretical XANES spectra of associative and dissociative PV2-xanthene complexes	**S15**
References	**S16**

Experimental:

Materials: Sodium vanadate ($NaVO_3$, 98% Sigma-Aldrich), sodium molybdate dihydrate ($Na_2MoO_4.2H_2O$, 99% Sigma-Aldrich), disodiumhydrogenphosphate (Na_2HPO_4, 99% Sigma-Aldrich), sulfuric acid (98% H_2SO_4, Sigma-Aldrich), vanadium (V) oxide (V_2O_5, Sigma-Aldrich, 99.6%), molybdenum trioxide (MoO_3, Sigma-Aldrich, 99.97%), dimethyl sulfoxide, DMSO (C_2H_6OS, Alfa Aesar, 99.8%), methanol (CH_3OH, VWR chemicals, 99.9%), deuterium oxide (D_2O, Sigma-Aldrich, 99.9%)

Synthesis of $H_4PV_1Mo_{11}O_{40}.nH_2O$ (n=24-32): 6.1 g (0.05 mmol) of $NaVO_3$ was dissolved in 100 mL of boiling water. Additionally, 7.1 g of Na_2HPO_4 was dissolved in 100 mL H_2O and added to the solution. After cooling the solution, 5 mL of concentrated sulfuric acid (98%) was added. 133.0 g (0.6 mmol) of $Na_2MoO_4.2H_2O$ dissolved in 200 mL of water was finally added to the solution and stirred for 15 minutes. 85 mL of concentrated H_2SO_4 (98%) was added dropwise to the solution and stirred vigorously. The dark red colored solution became light red after one hour. The $H_4PV_1Mo_{11}O_{40}.nH_2O$ was extracted with 400 mL of diethyl ether. The dark etherate layer in between water and ether was collected and dried in air to remove all the ether. The solution was then diluted with water and the $H_4PV_1Mo_{11}O_{40}.nH_2O$ was recrystallized and used as it is. Syntheses of $H_5PV_2Mo_{10}O_{40}.nH_2O$ and $H_6PV_3Mo_9O_{40}.nH_2O$ were performed by the same method by adjusting the ratio of $NaVO_3$ and $Na_2MoO_4.2H_2O$.

Characterization of $H_{3+x}PV_xMo_{12-x}O_{40}$ (x = 1–3). n H_2O (n=24-32): The characterization of the catalysts was carried out by ^{31}P as well as ^{51}V NMR. The ^{31}P and ^{51}V NMR were carried out on a Bruker Avance III 400 MHz and Bruker Avance NEO 400 MHz spectrometers.

HERFD XANES and XES measurements were conducted at the ID26 beamline of the European Synchrotron Radiation Facility (ESRF, Grenoble, France). The X-rays were generated by three mechanically independent undulators and monochromatized by a cryogenically cooled Si (311) double crystal monochromator. The emission spectrometer was equipped with five spherically bent (r = 1 m) Ge (422) analyzer crystals installed in a Rowland geometry and an avalanche photodiode detector. The counts of the detector were normalized by counts of a photodiode recording elastically scattered X-rays in front of the sample (I0). The resulting instrumental energy bandwidth was below 1.5 eV, which is comparable to the natural width of the V K edge core hole (1.01 eV). The beam size was maintained at 0.1 mm (vertical) × 0.2 mm (horizontal). Test scans with and without attenuators were recorded, and the corresponding temporal changes in the spectra were evaluated to identify the beam-induced changes in the samples (so-called “beam damage”). Due to significant beam damage an attenuator setting of 5 was used during the measurement. The X-ray absorption spectra in terms of HERFD-XANES were recorded by scanning the incident energy and detecting the fluorescence at the maximum of the V $K\beta_{1,3}$ emission line. Energy was calibrated using a vanadium metallic foil. The X-ray emission spectra around the main ($K\beta_{1,3}$) and the satellite ($K\beta''/K\beta_{2,5}$) emission lines were recorded between 5400 and 5490 eV while applying an excitation energy of 5600 eV.

The reference samples were measured in the form of pellets pressed with boron nitride as a binder (dilution to obtain max. 2 wt % V in the resulting mixture in order to mitigate incident beam self-absorption). The *in situ* spectra were collected by flowing the reaction mixture continuously through a Kapton tube (polyimide, outer diameter 3 mm, wall thickness 0.03 mm, Goodfellow) with the help of a peristatic pump. To prevent beam damage, a constant flow of 20 mL/min of the reaction mixture was maintained. However, even with a flow through a Kapton tube a gradual deposition of solid residue was observed on the inside surface of the Kapton tube wall at a spot where the incident X-ray beam hit the sample, with respective changes in the recorded data. To avoid this, the tube with the flowing sample was moved across the beam to an unexposed spot after every scan.

Data extraction and normalization (subtracting offset and normalizing to a new maximum to integrated area) were carried out in PyMCA software version 5.9.2.[1] The data alignment were carried out by measuring standard reference samples and also comparing to the previous measurements of the same beamline.[2] The presented HERFD-XANES spectra are averages of 10 scans normalized with default parameters in Athena v.0.9.26.[3]

Multivariate Curve Resolution Alternating Least Squares (MCR-ALS)[4] was performed in Fastosh v1.0.10[5] on the HERFD-XANES spectra normalized by subtracting minimum and taking the average intensity in the region 5520-5540 eV as unity. Please note that MCR-ALS was performed on the XES spectra without background subtraction as to avoid additional uncertainties which may come through the background fitting algorithm, normalization in this case was performed by subtract offset and normalizing per counts (integration using rectangles). SIMPLISMA was used for the initial estimation of spectral components, and non-negativity and closure constraints were applied to the concentration matrix, while only non-negativity constraint was applied to the spectral matrix. Three spectral components were suggested by the principal component analysis.

Calculation of theoretical XANES spectra: The theoretical XANES spectra at vanadium K-edge were calculated by the FEFF 10 code using multiple scattering theory.[6] DFT-optimized model structures were taken from literature[7] to generate the input files. The amplitude reduction factor, S_0^2, was set to 1, and the core hole was treated with random phase approximation. The radii for full multiple scattering (FMS) and self-consistent field (SCF) calculations were set to 5 Å.

Catalytic tests: All catalytic tests for diols and polyols oxidation were conducted in Fisher Porter tube or in an autoclave with a glass liner. The Fisher Porter tube was pressurized with 2 atm of air and the catalytic tests were run at 130^0C. The temperature was maintained with the help of an oil bath coupled to a thermocouple. For each catalytic test, 1 mmol of substrate and 0.1 mmol of catalysts were dissolved in 10 mL of reaction medium (1:1 water and methanol). After completion, the tube was cooled to room temperature and then placed in an ice bath for at least 1 hour for condensation of all the products formed. The reaction mixture was subsequently collected for analysis such as NMR. The mixture was stirred continuously during the reaction. For safety reasons, the Fisher Porter tube was protected with a cylindrical iron net.

The reaction between PV_x and xanthene was carried out in Fisher Porter tube under air or argon atmosphere. Typically, 1 mmol of xanthene and 2 mmol of PV_x (x= 1, 2, 3) were mixed in acetonitrile for the reaction under argon. For catalytic tests, 1 mmol of xanthene and 0.1 mmol of PV_x were mixed in acetonitrile. The reaction mixture was analyzed by gas chromatography.

Analysis of the products: The qualitative and quantitative analysis of all the reaction products were carried out by ^{1}H and ^{13}C NMR (Bruker Fourier 300 MHz) and gas chromatography (PerkinElmer, Clarus 580) with flame ionization detector. For ^{1}H NMR, a water suppression method was used as the peak of dimethoxymethane and methoxymethanol lies on the tail of the water peak. Known quantities of DMSO and the sodium salt of tetramethylsilane (TMS) were added calibration the peak and quantification of the products.

UV-Vis Spectroscopy: The ultraviolet visible absorption spectroscopy (UV-Vis) was recorded by mixing PVx (0.2 mmol in 50 mL acetonitrile) and xanthene (0.2 mmol in 50 mL acetonitrile) in a Shimadzu 1900i spectrophotometer in the range of 500-800 nm. We measured 2.5 mL of reaction mixture in a glass cuvette with an optical pathlength of 10 mm.

EPR Spectroscopy: We carried out the EPR experiments in a Bruker ELEXESYS 500 spectrometer in continuous wave in X band (approximately 9,5 GHz). Resonator was a 4119 HS high sensitivity resonator. EPR spectra was collected in the temperature range of 295-315 K with 10 K interval. For 10 dB attenuation, the power was 20 mW.

Table S1. ET-OT reaction between PV_x and xanthene. The product was quantified with gas chromatography. [(a)]1 mmol of xanthene and 2 mmol of PV_x were mixed in acetonitrile. [(b)]1 mmol of xanthene and 0.1 mmol of PV_x were mixed in acetonitrile for the reaction under Ar.

Catalyst	Time (h)	Temperature (°C)	Ar/Air	% Conversion (GC)	% Yield (GC)
PV1	8[(a)]	RT	Ar	3	-
PV2				23	-
PV3				43	-
PV1	18[(b)]	RT	Air	12	3
PV2				42	10
PV3				80	17
PV1	8[(a)]	RT	Air	5	1
PV2				13	2
PV3				20	4
PV1	18[(b)]	80	Air	73	42
PV2				80	50
PV3				98	72
PV1	18[(b)]	60	Air	70	23
PV2				79	40
PV3				94	67
PV1	24[(c)]	80	Air	95	92
PV2				97	95
PV3				97	96

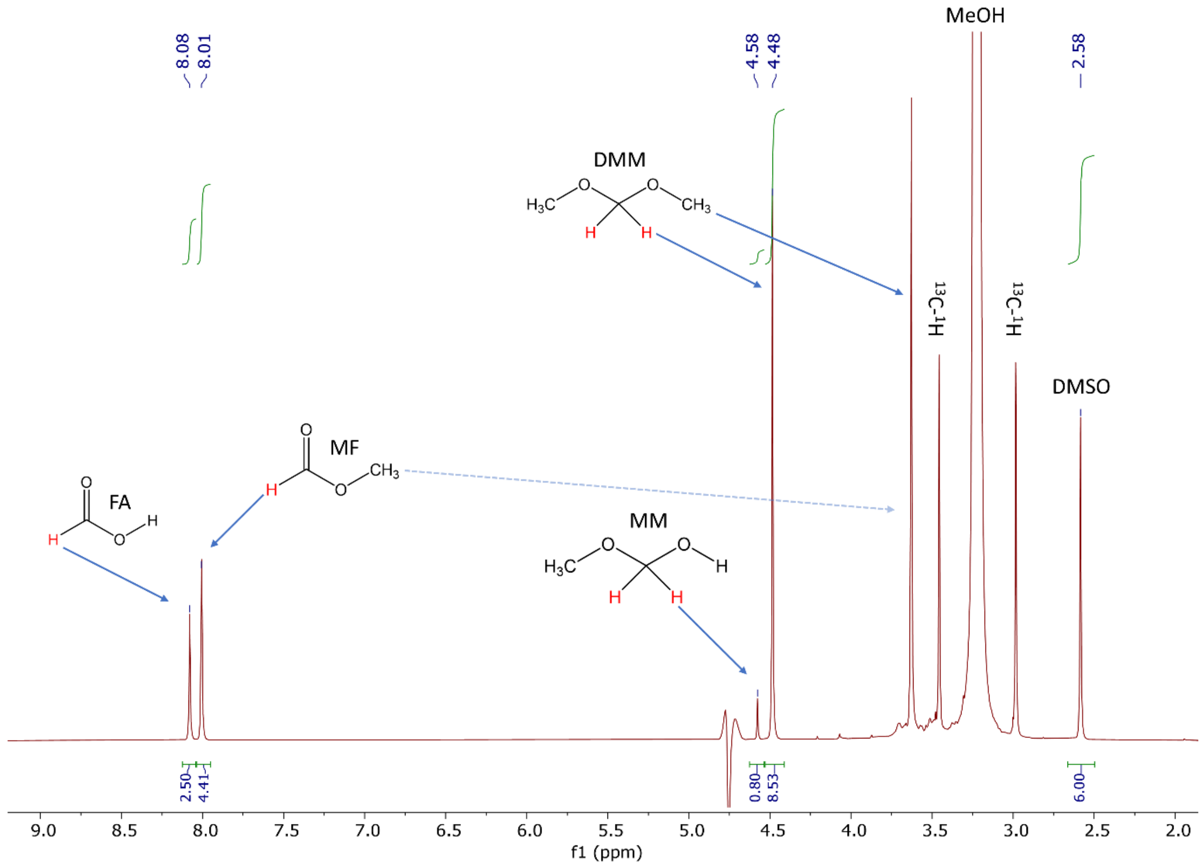

Figure S1. ^{1}H NMR of product mixture after reaction of PV2 with sorbitol. Reaction conditions: 1 mmol of sorbitol, 0.1 mmol of PV2, 10 mL of a 1:1 mixture of methanol and water, 130°C, 2 bar of air, 24 hours. Dry DMSO was added to quantify the product. The NMR was collected in water suppression mode.

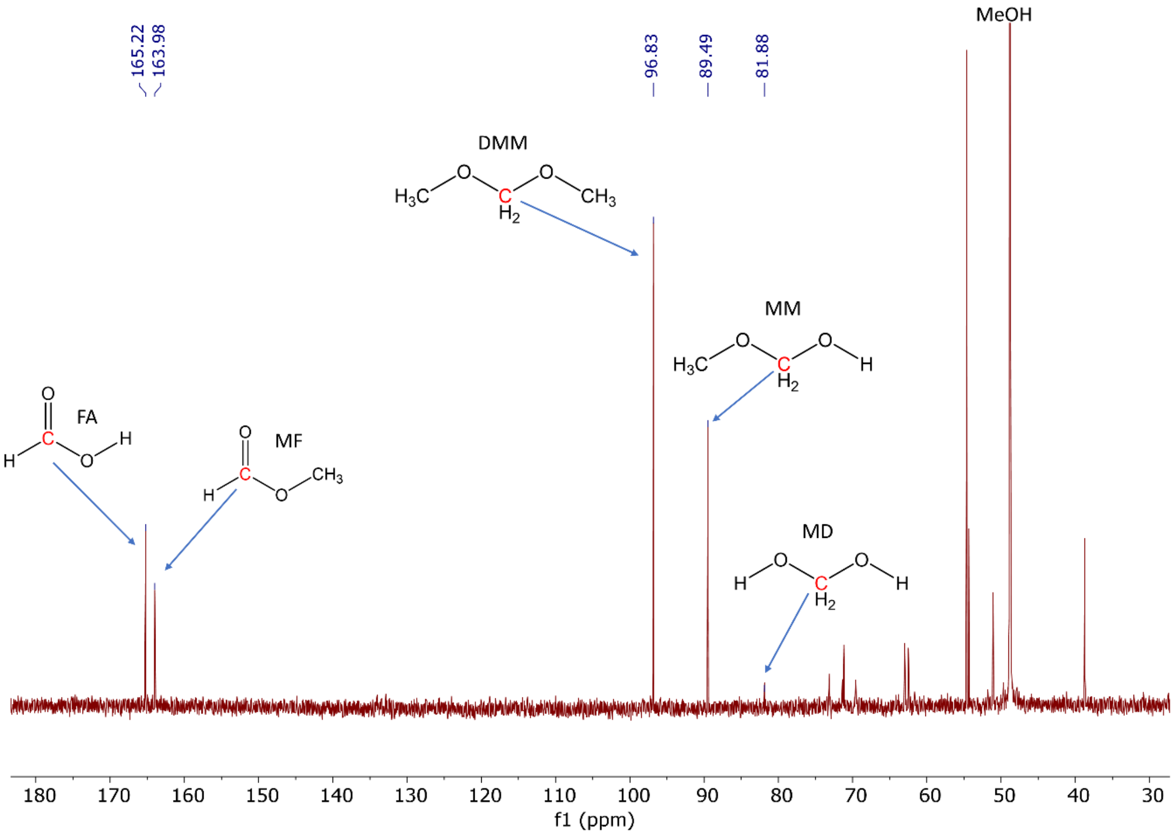

Figure S2. ^{13}C NMR of product mixture after reaction of PV2 with sorbitol. Reaction conditions: 1 mmol of sorbitol, 0.1 mmol of PV2, 10 mL of a 1:1 mixture of methanol and water, 130°C, 2 bar of air, 24 hours.

Table S2. Catalytic tests for oxidation of sorbitol for 24 hours. The products were quantified with ^{1}H NMR spectroscopy. [(a)]Glycerol, [(b)]Ethylene glycol, [(c)]Cellulose

Catalyst	T (^{o}C)	P (bar)	Solvent	Total Yield	% Selectivity			
					DMM	MM	FA	MF
V_2O_5	130	2	Water + Methanol	41	2	19	23	56
V_2O_4				9	5	19	27	49
V_2O_3				12	3	22	31	44
MoO_3				-	-	-	-	-
PV2			Water	46	-	-	100	-
PV2			Methanol	88	35	-	-	65
PV2[(a)]	150	5	Water + Methanol	13	62	-	18	20
PV2[(b)]				11	55	-	26	19
PV2[(c)]				14	63	-	17	20

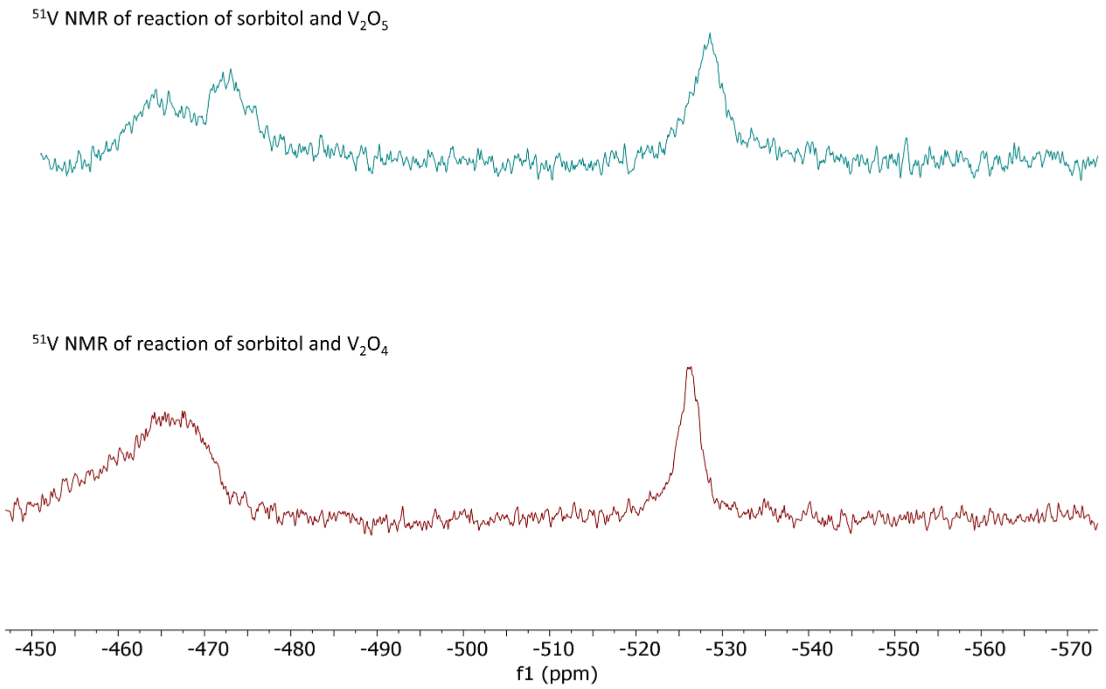

Figure S3. ^{51}V NMR spectra of filtered product mixture after reaction of V_2O_5 and V_2O_4 with sorbitol. Reaction conditions: 1 mmol of sorbitol, 0.1 mmol of V_2O_5 or V_2O_4, 10 mL of a 1:1 mixture of methanol and water, 130°C, 2 bar of air, 24 hours. The solution was filtered after the reaction and sorbitol was added to re-run the catalytic test. The product mixture was analyzed after the reaction. Molecular complexes of vanadium were found in the ^{51}V NMR spectra as shown above.

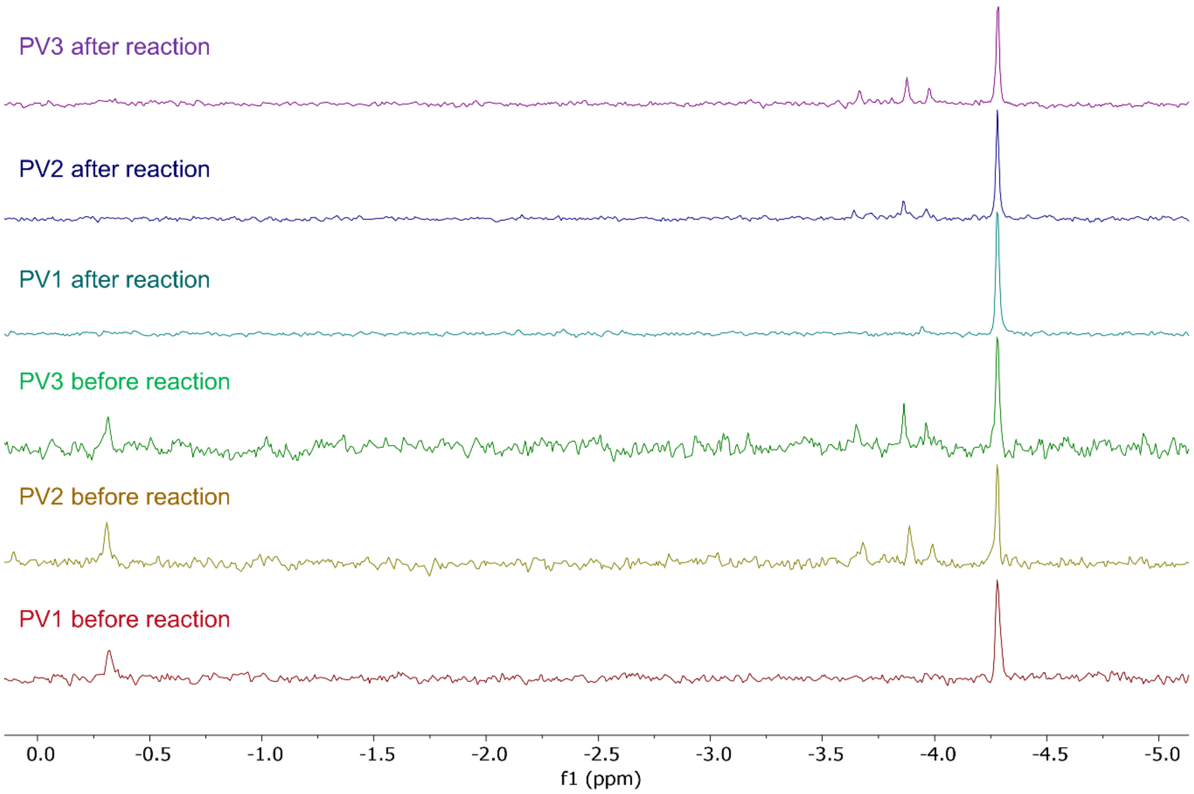

Figure S4. ^{31}P NMR spectra before and after reaction between PV_x and sorbitol. Reaction conditions: 1 mmol of sorbitol, 0.1 mmol of PV_x, 10 mL of a 1:1 mixture of methanol and water, 130°C, 2 bar of air, 24 hours.

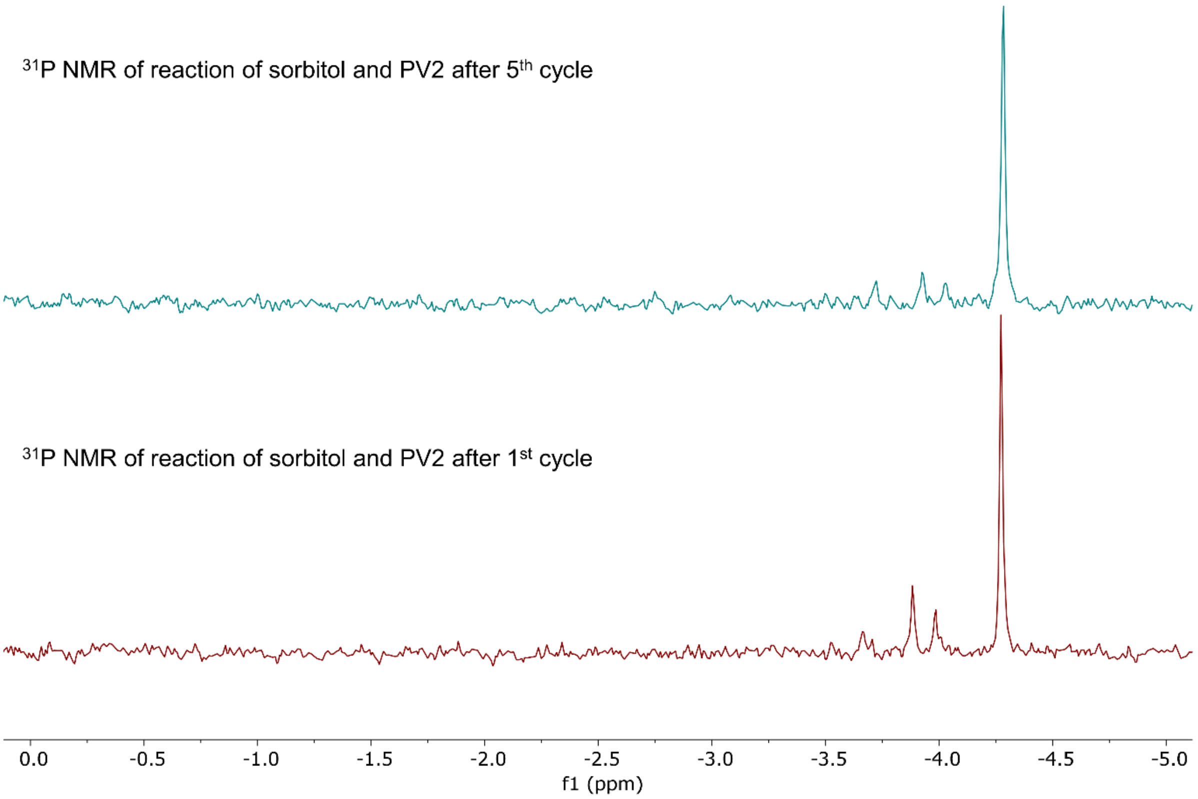

Figure S5. ^{31}P NMR spectra after first and five cycles of reaction between PV2 and sorbitol. Reaction conditions: 1 mmol of sorbitol, 0.1 mmol of PV2, 10 mL of a 1:1 mixture of methanol and water, 130°C, 2 bar of air, 24 hours. 1 mmol of substrate was added after every cycle.

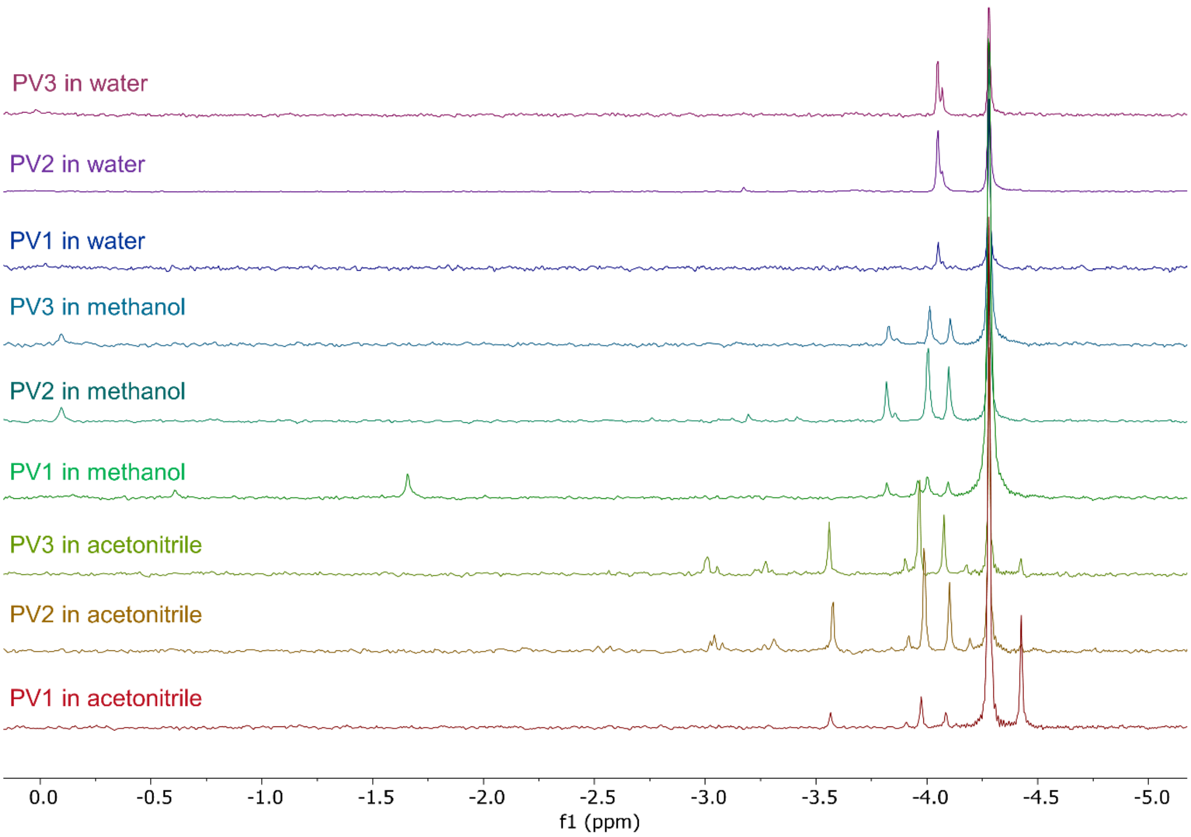

Figure S6. ^{31}P NMR spectra of PV1, PV2 and PV3 in water, methanol and acetonitrile. Different species were observed in different solvents which can result from coordination between solvent and PV1, PV2 and PV3.

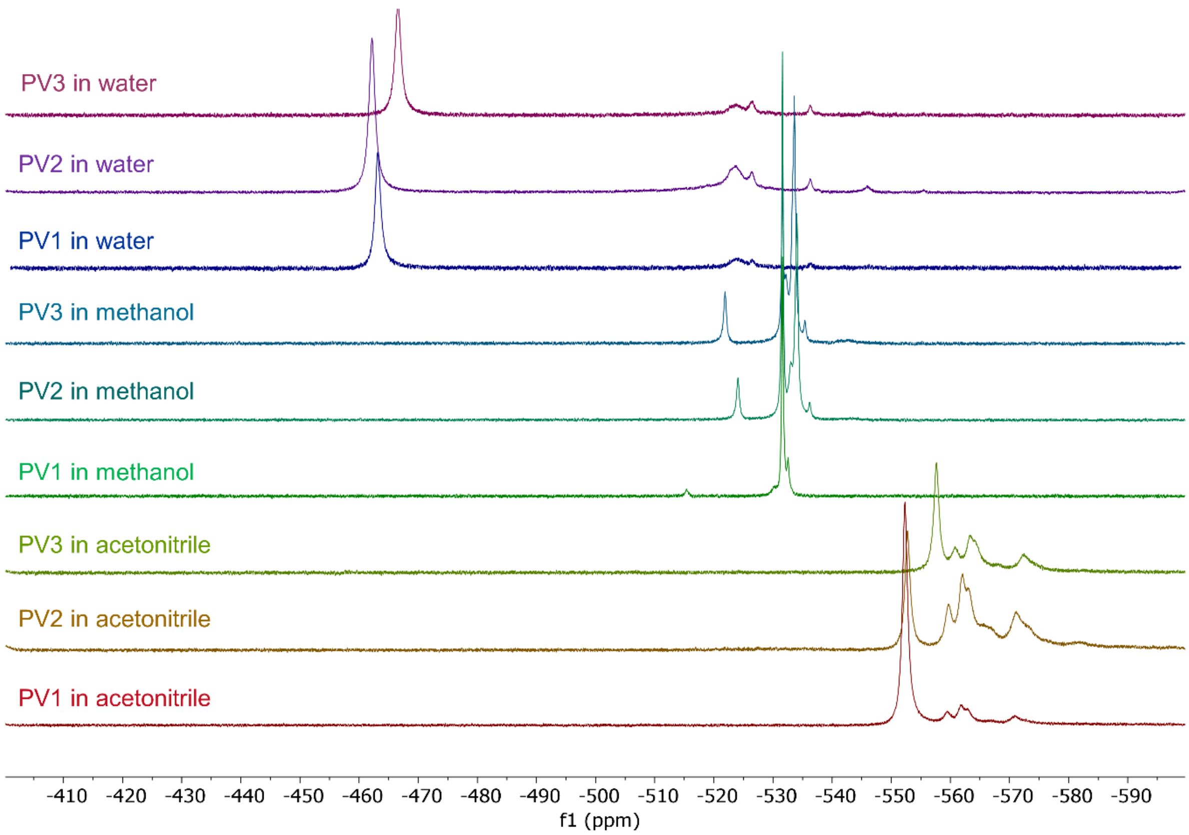

Figure S7. ^{51}V NMR spectra of PV1, PV2 and PV3 in water, methanol and acetonitrile. Different species were observed in different solvents which can result from coordination between solvent and PV1, PV2 and PV3.

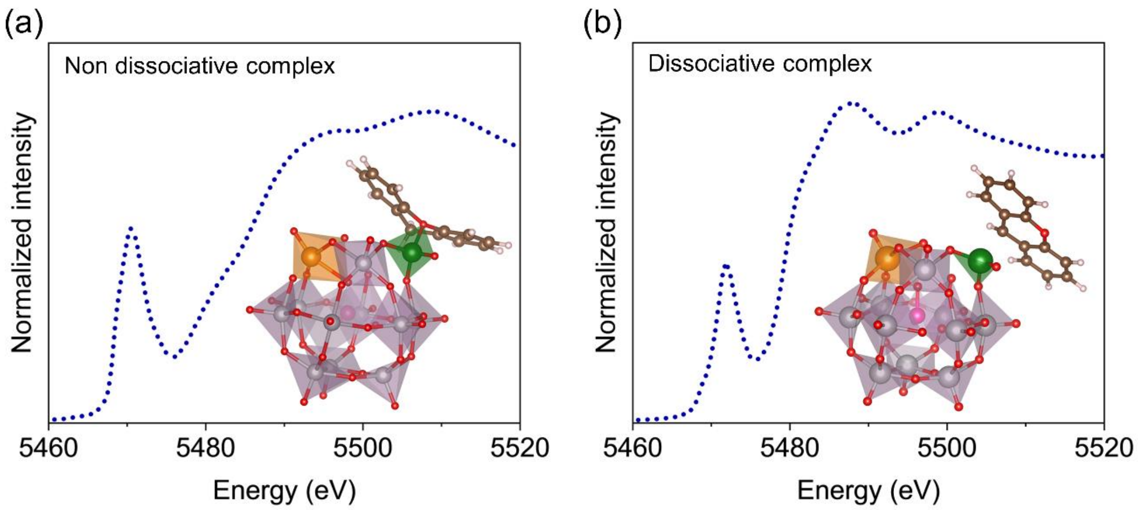

Figure S8. Theoretical XANES spectra of the PV2 with associative and dissociative complex with xanthene and the corresponding DFT optimized model structures (as represented in the fig. 6c of the manuscript) taken from reference.[7]

References:

1. V. A. Solé, E. Papillon, M. Cotte, P. Walter and J. Susini, *Spectrochimica Acta Part B: Atomic Spectroscopy*, 2007, **62**, 63-68.
2. D. E. Doronkin, F. Benzi, L. Zheng, D. I. Sharapa, L. Amidani, F. Studt, P. W. Roesky, M. Casapu, O. Deutschmann and J.-D. Grunwaldt, *The Journal of Physical Chemistry C*, 2019, **123**, 14338-14349.
3. B. Ravel and M. Newville, *J Synchrotron Radiat*, 2005, **12**, 537-541.
4. J. Jaumot, R. Gargallo, A. de Juan and R. Tauler, *Chemometrics and Intelligent Laboratory Systems*, 2005, **76**, 101-110.
5. G. Landrot and E. Fonda, *Journal of Synchrotron Radiation*, 2025, **32**, 1085-1094.
6. J. J. Rehr and R. C. Albers, *Reviews of Modern Physics*, 2000, **72**, 621-654.
7. I. Efremenko and R. Neumann, *Journal of the American Chemical Society*, 2012, **134**, 20669-20680.
